# Prevalence of Emergence Delirium and Associated Factors among Older Patients Who Underwent Elective Surgery: A Multicenter Observational Study

**DOI:** 10.1155/2022/2711310

**Published:** 2022-09-09

**Authors:** Gezahegn Tesfaye Mekonin, Megersa Kelbesa Olika, Mitiku Birhanu Wedajo, Ashanafi Tolasa Badada, Abebe Dukessa Dubiwak, Tajera Tageza Ilala, Mamo Nigatu Gebre

**Affiliations:** ^1^Department of Anesthesia, Institute of Health, Jimma University, Jimma, Ethiopia; ^2^Department of Biomedical Science, Faculty Medical Science, Institute of Health, Jimma University, Jimma, Ethiopia; ^3^Department of Anesthesia, College of Medicine and Health Science, Hawassa University, Hawassa, Ethiopia; ^4^Department of Epidemiology, Institute of Health, Jimma University, Jimma, Ethiopia

## Abstract

**Background:**

Emergence delirium is a common and serious postoperative complication in older surgical patients. It occurs at any time in the perioperative period, during or immediately following emergence from general anesthesia. Unfortunately, it is highly associated with postoperative complications such as a decrease in functional capacity, prolonged hospital stay, an increase in health care costs, and morbidity and mortality. The goal of this study was to determine the prevalence of emergence delirium and associated factors among older patients who underwent elective surgery in the teaching hospitals of Ethiopia at the postanesthesia care unit in 2021.

**Methods:**

A multicenter prospective observational study was conducted at the postanesthetic care unit in the four teaching hospitals of Ethiopia. Older surgical patients admitted to the postanesthesia care unit who underwent elective surgery in the four teaching hospitals of Ethiopia were selected by using simple random sampling. Pretested structured questionnaire was used to collect data. Data were entered into EpiData (version 4.6) and exported to the SPSS (version 25.0). Binary logistic regression was used to identify factors independently associated with the emergence delirium.

**Results:**

Out of 384 older patients included in the study, the prevalence of emergence delirium was 27.6%. Preoperative low hemoglobin levels (AOR: 2.0, 95% CI; 1.77–3.46), opioid (AOR: 8.0, 95% CI; 3.22–27.8), anticholinergic premedications (AOR: 8.5, 95% CI; 6.85–17.35), and postoperative pain (AOR: 3.10, 95 CI; 2.07–9.84) at PACU were independently associated with emergence delirium.

**Conclusion:**

The prevalence of emergence delirium was high among older patients who underwent elective surgery. Opioid and anticholinergic premedication, low preoperative hemoglobin, and the presence of postoperative pain were independently associated with the emergence delirium. Adequate preoperative optimization and postoperative analgesia may reduce the prevalence of emergence delirium.

## 1. Introduction

Emergence delirium is defined as a state of acute restlessness and confusion manifested by changes in the mental state, loss of attention, and disorganized thinking [1, 2].

It may be manifested by acute deterioration in attention and cognition, which may include changes in levels of consciousness and thinking [3, 4]. It could occur in the operation room or PACU at any time during or immediately following emergence from general anesthesia [1–3].

It is a common complication in surgical patients during the early postoperative period, complicating up to 87% of critically ill patients; 10–24% of general adult medical patients; 37–46% of general surgical patients receiving anesthesia, and in up to 50% of older surgical patients during early emergence at PACU [4–6]. Hence, its prevalence varies significantly depending on the type of procedure and age of the patients; being old is the most vulnerable age group [2, 5, 6].

It varies according to the patient population, the type and urgency of surgery, and the type and sensitivity of assessment tools. Hence, it is a major adverse event that causes considerable distress to patients, families, and health professionals in the postoperative period [4, 7–9].

Emergence delirium possesses a serious risk to the patient and PACU staff like violent behavior leading to injury, disrupting wound dressing, increased pain, hemorrhage, and self-removal of medical devices (like endotracheal tubes, catheters, and drainage tubes) with increased health care expenditure and postoperative mortality placing difficulties in giving patient care [5, 10, 11].

Certain preoperative medication such as anticholinergics and benzodiazepines, as well as the history of chronic smoking and alcohol abuse, has been independently associated with the experience of emergence delirium [12–14].

Prevention and treatment of emergence delirium need a multidisciplinary team approach. Hence, it comprises risk assessment and stratification, risk reduction, and early identification and management. Prevention is the most effective management of delirium [15, 16].

It is suggested that nonpharmacological therapy could be applied to control risk factors like sleep problems, immobility, sensory deficits, pharmacological therapy, and adequate hydration to combat dehydration, which has been indicated in reducing the risk of delirium [17].

It is challenging in some clinical situations and patients to diagnose and treat emergence delirium such as in the presence of anxiety and emotional disturbance that makes the treatment of some patients difficult. However, such conditions might need conscious sedation with benzodiazepines like midazolam [18], as to decrease or abolish the physiological and psychological effects for effective prevention and management of these conditions in some clinical areas like the dental procedures, or other ambulatory setup. Thus, it might interfere with the early diagnosis and intervention of delirium [19].

It is a common perioperative problem in older patients that we encounter after anesthesia and surgery at PACU. Particularly, surgical teams including anesthetists, surgeons, nurses, residents, interns, and PACU nurses were encountering emergence delirium in older patients. As long as an additional burden on manpower is considered, there exists a need for an increase in the number of health professionals caring for delirious patients. Especially, PACU staff must always stay by the patient's side. However, while nurses, medical interns, or other professionals are caring for delirious patients, nondelirious patients might be less closely monitored, thereby reducing their care and increasing their anxiety. Therefore, having noticed the prevalence of emergence delirium, identifying the associated factors is crucial to avoiding the aforementioned complications. Thus, we aimed to determine the prevalence of emergence delirium and its associated factors in older patients who underwent elective surgery in teaching hospitals in Ethiopia at PACU.

The objective of this study is to assess the prevalence of emergence delirium and associated factors among older patients who underwent elective surgery in the Teaching Hospitals of Ethiopia, 2021.

## 2. Methods and Materials

### 2.1. Study Area, Study Design, Study Period, and Population

A multicenter prospective observational study design was conducted in the four teaching hospitals of Ethiopia from February 05 to March 30, 2021, at the postanesthesia care unit. The teaching hospitals of Ethiopia were serving as a center of teaching for medical students and bear different clinical care delivery, and also an academic upgrading or specialty. Older patients ≥60 years, who underwent elective surgery during the study period, were included. However, unconscious patients at PACU, patients with preexisting complicated comorbid diseases, and/or psychiatric problems like dementia were excluded.

The study was published as a preprint in the research square with the unique registration number rs-1719192 [20].

### 2.2. Operational Definition

Confusion Assessment Method–Intensive Care Unit (CAM-ICU) was used to diagnose emergence delirium. Accordingly, the presence of feature 1 (acute onset and fluctuating course), feature 2 (inattention), and either feature 3 (disorganized thinking) or feature 4 (altered level of consciousness) within the first one hour was considered as emergence delirium. CAM-ICU is easy to administer, quick with good specificity and sensitivity for the diagnosis of emergence delirium in older patients who were awake, not critically ill, and mechanically ventilated, and it enables assessment of the cognitive function as well [21–25].

Postoperative sedation (consciousness) and agitation were assessed by the Richmond Agitation Sedation Scale (RASS), a 10-point scale between +4 and −5 [24, 26, 27].

Patients who respond to verbal commands (RAAS score of other than −4 or −5 as described below) were evaluated for delirium by CAM: 4-combative (violent, immediate danger to his/her self or staff), 3-very agitated (pulls or removes tube or catheters, aggressive), 2-agitated (frequent nonpurposeful movements, fight), 1-restless (anxious, but no aggressive movements), 0-alert and calm, −1-drowsy (sustained awakening to voice (>10 sec)), −2-light sedation (briefly awakens with eye contact to voice (<10 sec)), −3-moderate sedation (movement or eye-opening to voice, but no eye contact), −4-deep sedation (no response to voice, but movement or eye-opening to physical stimulation), and −5-unarousable (no response to voice or physical stimulation).

Postoperative patient pain was assessed by using a numeric rating score, which had a score of 0–10, with 0–3 = no pain–mild pain, 4–6 = moderate pain, and 7–10 = severe pain.

Anemia is a clinical syndrome defined by low hemoglobin level; hemoglobin levels <12.0 g/dL in women and hemoglobin levels <12 g/dL in men are considered anemia [28].

Older patients are the patient population who were 60 and over years old [27].

### 2.3. Sample Size Determination and Sampling Techniques

#### 2.3.1. Sample Size Determination

The sample size was calculated by the single population proportion formula, using a 50% proportion of the older population with emergence delirium, 95% CI, and a margin of error of 5%.

(1) $$\begin{array}{c} n=\frac{\left(z\alpha /2\right)2pq}{d2}, \end{array}$$

where *n* = number of sample size, *Zα*/2 at 95% CI = 1.96, *p* = proportion of emergence delirium, 50%, *q* = 1 − p; for this study, maximum variability is presumed; hence, *p*=0.5; *q* = 1 − *p* = 0.5, *d* = level of precision at 95% confidence level, that is, 5% of sample size = 0.05, by incorporating into the formula; *n* = (1.96) 2 (0.5) (0.5)/(0.05) 2 = 384. Hence, a sample size of 384 was obtained.

#### 2.3.2. Sampling Techniques

Multistage sampling techniques were implemented in the four teaching hospitals of Ethiopia including Hawassa University Comprehensive Specialized Hospital, Jimma Medical Center, Tikur Anbessa Teaching and Specialized Hospital, and Ambo Referral Hospital. The annual admission to PACU was obtained at each selected teaching hospital. The sample size was allocated proportionally for each selected hospital based on each hospital's annual older patients PACU admission. Accordingly, the sample size was 149, 123, 100, and 12, which were taken from Tikur Anbessa Hospital, Jimma Medical Center, HUCSH, and Ambo Referral Hospital, respectively.

Based on the daily operation schedule list at each hospital, older patients were selected using simple random sampling. The selected patients, who were admitted to PACU in the study period, were included.

### 2.4. Data Collection Instruments and Techniques

Data were collected using a structured questionnaire. We carried out the COVID-19 prevention strategy. At patient admission to PACU, the sociodemographics, preoperative, and intra- and postoperative factors were reviewed and recorded. Data collection procedures included a review of patient records, an operation note and anesthesia chart, an interview with the patient, and direct observation of the patient's RASS score and CAM score. For data quality, 20 patients (5% of the sample size) were pretested at PACU, and possible modifications were made. Regular monitoring and follow-up were made during data collection.

### 2.5. Data Management and Analysis

The data were entered into the EpiData (version 4.6.0.6) and exported to the SPSS windows statistical software (version 25.0) for data processing and further statistical analysis. Bivariable logistic regression was used to select candidate variables for multivariable logistic regression. Variables with a *p*-value less than 25% in the bivariable logistic regression were a candidate for the multivariable logistic regression. Multivariable logistic regression was fitted to identify factors independently associated with the emergence delirium and to control confounders. In the final model, AOR and 95% CI were used to measure the strength of association and statistical significance respectively.

### 2.6. Ethical Consideration

The ethical clearance letter was obtained from Jimma University Institutional Review Board, and verbal informed consent was obtained from each study participant. Confidentiality of the study participant was maintained. We applied the WHO COVID-19 prevention strategy.

## 3. Results

### 3.1. Sociodemographic Characteristics

A total of 384 patients were included in the study with a 100% response rate. Greater than half (57.8%) of the study participants were males, and the rest, 162 (42.2%), were females. Regarding the age of the participants, 170 (44.3%) of the participants were between 65 and 74 years. The majority, 312 (81.3%), of the participants were married. With respect to educational status, greater than one-third (37.2%) of the study participants had no formal education.

### 3.2. Preoperative Factors

As the distribution of preoperative factors showed, ASA II held nearly three-fourths (74.2%) of the ASA status of older patients, while ASA-I and ASA-III were 84 (21.9%) and 15 (3.9%), respectively. Those patients who had a history of comorbid disease were 76 (19.8%). Opioids were the most frequently administered premedication, 120 (31.3%), followed by anticholinergic, 105 (27.3%), and other premedication like diazepam and steroids, 95 (24.7%). Regarding preoperative hemoglobin levels, more than three-fourths (81.0%) of the older patients have normal hemoglobin levels, and the rest, 19.0%, have low hemoglobin levels (anemic) (Table 1).

### 3.3. Intraoperative and Postoperative Factors

General anesthesia was provided to nearly more than half of the older patients (54.2%). 85 (39.9%) of older patients were induced with ketofol, and the majority (86.4%) of older patients were maintained with inhalation anesthetics, with isoflurane accounting for nearly two-thirds (67.9%) of the participants. The majority of surgeries were longer than two hours, with 314 (81.8%) taking a long time (Table 2).

**Figure 1:**
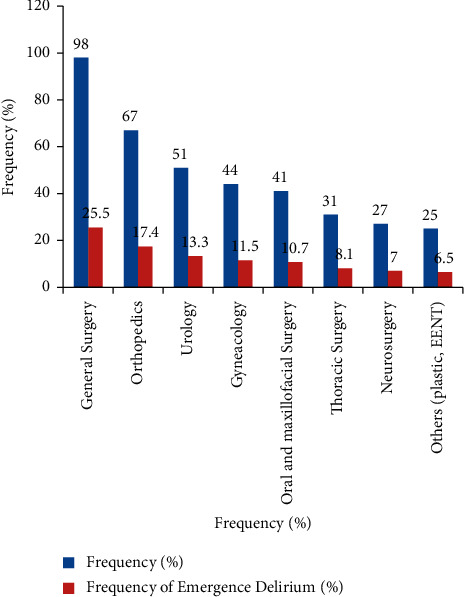
The frequency of types of surgery done and the prevalence of emergence delirium in older patients who underwent elective surgery at teaching hospitals of Ethiopia.

General surgery was the most commonly performed type of surgery among older patients as compared to other specialties, accounting for 98 (25.5%) of the total, as shown in the bar chart (Figure 1).

**Figure 2:**
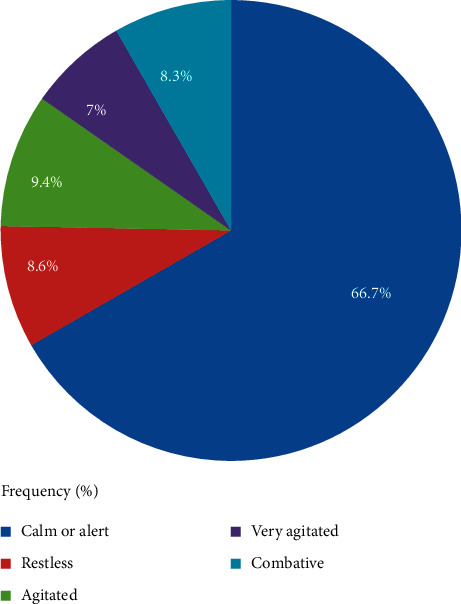
The frequency of sedation score in older patients who underwent elective surgery in the Teaching Hospitals of Ethiopia.

Two-thirds (66.7) of older patients were calm, and the rest were agitated, 36 (9.4%), restless, 33 (8.6%), combative, 32 (8.3%), and very agitated, 27 (7.0%), respectively, according to the PACU sedation score, as shown in Figure 2.

### 3.4. Prevalence of Emergence Delirium

The prevalence of emergence delirium in older patients who underwent elective surgery at the teaching hospitals of Ethiopia was 106 (27.6%).

#### 3.4.1. Factors Associated with Emergence Delirium among Older Patients Who Underwent Elective Surgery at PACU

Bivariable and multivariable logistic regression of factors are associated with the emergence delirium in older patients who underwent elective surgery at Teaching Hospitals of Ethiopia.

Bivariable logistic regression was fitted to identify candidate variables for the multivariable logistic regression. Accordingly, educational status, marital status, premedication with opioids and anticholinergic, comorbidity, preoperative hemoglobin level, types of anesthesia, maintenance agent, postoperative pain, and postoperative sedation were associated with the emergence delirium at a *p*-value of <0.25 (Table 3).

#### 3.4.2. Factors Independently Associated with the Emergence of Delirium in Older Patients Who Underwent Elective Surgery at Teaching Hospitals of Ethiopia

Multivariable logistic regression was fitted to identify factors independently associated with the emergence delirium. Accordingly, preoperative low hemoglobin levels, premedication of opioids and anticholinergic, and moderate to severe postoperative pain at PACU were significantly associated with the emergence delirium in multivariable logistic regression at a *p*-value of <0.05.

When compared to older patients who did not receive opioid premedication, those who received opioid premedication were 8 times (AOR: 8.0, 95% CI: 3.22–27.8), more likely to suffer the emergence delirium. Anticholinergic premedication increased the risk of the emergence delirium in older patients by 8.5 times (AOR: 8.5, 95% CI: 8.5 (6.85–17.35) as compared to those who had not.

When compared to older patients with normal preoperative hemoglobin, those with low preoperative hemoglobin were 2 times (AOR; 2.0, 95% CI, 1.77–3.46) more likely to suffer the emergence delirium. When compared to older patients experiencing mild pain in the PACU, those experiencing moderate to severe pain were 3.10 times (AOR; 3.10, 95% CI, 2.07–9.84) more likely to develop the emergence delirium (Table 4).

## 4. Discussion

In this study, the overall prevalence of emergence delirium among older patients who underwent elective surgery in the teaching hospitals of Ethiopia was 27.6%. An observational study conducted in China showed that the prevalence of emergence delirium among older patients at PACU was 37.0%. Another observational study conducted at the University of Gondar Hospital, Ethiopia, found that the prevalence of emergence delirium at PACU among older patients who underwent surgical procedures was 40.7%. Our finding is relatively low as compared to the previous study. This variation might be explained by the inclusion of patients who underwent emergency surgery (which had a high prevalence of emergence delirium as it permits limited time to optimize patients operated in an emergency setting) and might be due to the large sample size used in the previous study and tool difference [23, 27].

A prospective study carried out in Thailand and a retrospective study done in Korea showed that the prevalence of emergence delirium among older patients was 11.6% and 18.3%, respectively. Our finding is relatively high as compared to the previous study. A possible explanation for this discrepancy might be due to inadequate preoperative optimization and reassurance of the patient, inadequate postoperative pain management in the present study, and the clinical setup differences [26, 29].

In our study, older patients who had been given opioids premedication were 8 times (AOR: 8.0, 95% CI: 3.22–27.8) more likely to develop emergence delirium as compared to those who were not given opioids premedication. This is in corroboration with the study conducted in China that agreed that those older surgical patients who had taken perioperative opioids were incredibly suffering from emergence delirium. Similarly, a study was conducted in Gondar that agreed that older patients who had taken perioperative opioids were 5 times (AOR: 5.0, 95% CI: 1.265–20.565) more likely to develop emergence delirium as compared to those who had not taken intravenous opioids. Another study asserted that older patients who had pain management with opioids were more likely to develop emergence delirium as compared to those who had managed with nonopioid analgesics. This finding is supported by another study that argued that opioids increase the likelihood of adverse outcomes such as delirium. This variation might be explained as there exists increased drug sensitivity in older patients due to the dramatic changes in receptor function, which heightened the sensitivity of the brain toward adverse effects of opioids leading to delirium [23, 27, 30, 31].

Older patients who had anticholinergic premedication were 8.5 times (AOR: 8.5, 95% CI: 8.5 (6.85–17.35) more likely to develop emergence delirium as compared to those who were not given anticholinergic premedication. The finding of our study is in agreement with a study conducted in Australia, which contended that older patients who had taken anticholinergic medication were significantly associated with an increased risk of experiencing emergence delirium. Another study conducted in India argued that an anticholinergic medication like atropine was significantly associated with the emergence of delirium. The possible explanation might be due to anticholinergic medication blocking cholinergic transmission on the postsynaptic muscarinic receptors (M1 receptors), which were primarily located in the central nervous systems and involved in perception, attention, and cognitive function [32, 33].

Older patients who had low preoperative hemoglobin were 2 times (AOR; 2.0, 95% CI, 1.77–3.46) more likely to develop the emergence delirium as compared to those who had normal hemoglobin. A similar finding was observed in an observational study conducted in Germany, which ascertained that older patients who had low hemoglobin in the postoperative period were 4 times (AOR; 4.0, 95% CI, 1.36–11.48) more likely to develop postoperative delirium as compared to older patients who had normal hemoglobin levels. Another study found that older patients with more frequent low preoperative hemoglobin levels experienced delirium. The possible explanation for this might be due to low hemoglobin levels limiting the cerebral oxygen delivery; in a sense, inadequate cerebral oxygen supply with increased metabolic demand resulted in an imbalance of neurotransmitters, the disintegration of blood-brain barriers, and subsequent neuroinflammation [24, 28].

In our study, older patients who had postoperative pain (Numeric Rating Scale > 5) at PACU were 3.10 times (AOR; 3.10, 95% CI, 2.07–9.84) more likely to develop the emergence delirium as compared to those who had no pain. Our finding is supported by a study conducted in Korea asserted that patients with pain (NRS ≥ 6) were 3.6 times more likely to develop emergence delirium, and more than half of those patients with the emergence delirium were reported to have severe immediate postoperative pain at PACU. Comparably, a study was conducted in Gondar that extrapolated that emergence delirium was more likely when the pain numeric rating scale was greater than or equal to five (NRS ≥ 5). This was argued by another study, which concluded that inadequate analgesia in the perioperative period had been strongly associated with the emergence delirium. The possible explanation for this might be due to the perceived psychological effects of pain, and pain may favor alterations in the neurotransmitter systems inducing the proinflammatory mediators and impairing the physiological stress response resulting in neurophysiological dysfunctions that may potentiate cognitive impairments that may be manifested as delirium [10, 27, 34].

### 4.1. Strength and Limitation of the Study

To the best of our knowledge, this is one of the interesting studies to investigate the prevalence of emergence delirium and associated factors among older patients who underwent elective surgery at PACU. This study will primarily maximize the older patient's safety and reduce the prevalence of emergence delirium among older patients who underwent elective surgery.

The limitations of our study were ascertained as follows: we did not assess emergence delirium among older patients after PACU discharge or during the hospital stay and the long-term outcome of emergence delirium. Therefore, we had no remark on the continued effects of emergence delirium beyond the PACU in the postoperative period and the consequence it may have on patient outcomes.

## 5. Conclusion and Recommendation

The prevalence of emergence delirium among older patients who underwent elective surgery in the teaching hospitals in Ethiopia was high. Thus, the multidisciplinary teams approach is required including both the medical and surgical teams to prevent and manage the emergence delirium during the perioperative period.

The administration of preoperative anticholinergic and opioids was independently associated with the emergence delirium. Thus, health care provider's including the anesthesia teams, postanesthesia care nurses, interns, and other health care providers should emphasize preoperative optimization and reduction or, if possible, omit opioids and anticholinergic premedication in older patients.

Low preoperative hemoglobin was independently associated with the emergence delirium. Hence, surgical teams and anesthesia providers should optimize patients with low preoperative hemoglobin.

Postoperative pain at PACU was independently associated with the emergence delirium. Anesthetists, PACU teams, and other health care providers giving care for the patient should ensure adequate postoperative analgesia with wise selection and dosing of analgesics (particularly, those devoid of opioid side effects), which may reduce the prevalence of emergence delirium.

In general, perioperative handling including prevention and management of emergence delirium needs multidisciplinary teams in the risk removal, risk reduction, and stratification. Furthermore, the early diagnosis and management of emergence delirium are a crucial point behind reducing its prevalence and complication.

## Figures and Tables

**Table 1:** A cross-tabulation of the preoperative factors in older patients who underwent elective surgery in the teaching hospitals of Ethiopia, Ethiopia (*n* = 384).

Variables	Categories		Frequency (%)	Emergence delirium, frequency (%)	
				Yes (*n* = 106)	No (*n* = 278)
				106 (27.6)	278 (72.4)
Patient ASA status	ASA-1		84 (21.9)	25 (29.8)	59 (70.2)
	ASA-2		285 (74.2)	73 (25.6)	112 (74.4)
	ASA-3		15 (3.9)	8 (53.3)	7 (46.7)

Comorbidity	Yes		76 (19.8)	35 (46.1)	41 (53.9)
	No		308 (80.2)	71 (23.1)	237 (76.9)

Premedication	Anticholinergics	Yes	105 (24.7)	57 (60)	48 (40)
		No	289 (75.3)	49 (17.0)	240 (83.0)
	Opioids	Yes	120 (31.3)	65 (54.2)	55 (45.8)
		No	264 (68.8)	41 (15.5)	223 (84.5)
	Others (steroids, diazepam)	Yes	95 (27.3)	37 (35.2)	58 (64.8)
		No	279 (72.7)	69 (24.7)	210 (75.3)

Preoperative Hgb levels	Anemic		73 (19.0)	61 (83.6)	12 (16.4)
	Nonanemic		311 (81.0)	45 (14.5)	266 (85.5)

**Table 2:** A cross-tabulation of the intraoperative and postoperative factors in older patients who underwent elective surgery at Teaching Hospitals of Ethiopia, Ethiopia (*n* = 384).

Variables	Categories	Frequency (%)	Emergence delirium, frequency (%)	
			Yes (*n* = 106)	No (*n* = 278)
			106 (27.6)	278 (72.4)
Types of anesthesia	Regional anesthesia	176 (45.8)	34 (19.3)	142 (80.7)
	General anesthesia	208 (54.2)	72 (34.6)	136 (65.4)

Induction agent	Ketamine	28 (13.1)	10 (35.7)	18 (64.3)
	Propofol	62 (29.1)	25 (40.3)	37 (59.7)
	Thiopental	38 (17.9)	14 (36.8)	24 (63.2)
	Ketofol	85 (39.9)	29 (34.1)	56 (65.9)

Maintenance agent (s)	Inhalational	184 (86.4)	73 (39.7)	111 (60.3)
	Propofol	3 (1.4)	1 (33.3)	2 (66.7)
	Others	26 (12.2)	4 (15.4)	22 (84.6)

Inhalational agent for maintenance	Halothane	68 (32.1)	34 (50)	34 (50)
	Isoflurane	144 (67.9)	44 (30.6)	100 (69.4)

Duration of surgery	Short duration (<2 hours)	70 (18.2)	16 (22.9)	54 (77.1)
	Longer duration (>2 hours)	314 (81.8)	90 (28.7)	224 (71.3)

Postoperative pain	No-mild	362 (94.3)	93 (25.7)	269 (74.3)
	Moderate-severe	22 (5.7)	13 (59.1)	9 (40.9)

**Table 3:** Bivariable logistic regression of factors associated with the emergence delirium in older patients who underwent elective surgery in the teaching hospitals of Ethiopia, Ethiopia (*n* = 384).

Variables	Categories		Emergence delirium, frequency (%)		COR (95% CI)	*p*-value
			Yes (*n* = 106)	No (*n* = 278)		
			106 (27.6)	278 (72.4)		
Educational status	High education		26 (22.6)	89 (77.4)	1	
	Low education		18 (14.3)	108 (85.7)	0.57 (0.22–0.66)	**0.22**
	No		62 (43.4)	81 (56.6)	2.62 (0.90–3.40)	**0.18**

Marital status	Married		71 (22.8)	241 (77.2)	1	
	Single		23 (46.9)	26 (53.1)	3.0 (0.46–3.33)	**0.22**
	Divorced		12 (52.2)	11 (47.8)	4.16 (1.57–8.75)	**0.23**

Comorbidity	No		71 (23.1)	237 (76.9)	1	
	Yes		35 (46.1)	41 (53.9)	2.85 (2.11–10.0)	**0.16**

Preoperative hemoglobin level	Nonanemic		45 (14.5)	266 (85.5)	1	
	Anemic		61 (83.6)	12 (16.4)	30.1 (3.10–26.0)	**0.24**

Types of anesthesia	Regional anesthesia		34 (19.3)	142 (80.7)	1	
	General anesthesia		72 (34.6)	136 (65.4)	2.21 (1.82–9.2)	**0.22**

Premedication	Anticholinergic	No	49 (17)	240 (83)	1	
		Yes	57 (60)	38 (40)	7.35 (5.5–11.3)	**0.12**
	Opioids	No	41 (15.5)	223 (84.5)	1	
		Yes	65 (54.2)	55 (45.8)	6.43 (20–55)	**0.23**

Types of surgery	Others (Plastics and EENT)		2 (7.4)	23 (92.6)	1	
	Urology		8 (19.5)	33 (80.5)	2.79 (1.21–9.33)	0.44
	Neurologic surgery		10 (37.0)	17 (63.0)	6.76 (2.0–17.15)	**0.23**
	Oral and maxillofacial		10 (19.6)	41 (80.4)	2.80 (0.42–8.55)	0.26
	Gynecology		10 (22.7)	34 (77.3)	3.38 (1.06–12.0)	0.54
	Thoracic		13 (41.9)	18 (58.1)	8.31 (4.44–21.1)	**0.19**
	Orthopedics		18 (27.7)	49 (72.3)	4.22 (0.65–3.88)	**0.20**
	General surgery		35 (35.7)	63 (64.3)	6.39 (1.46–17.2)	**0.22**

Postoperative pain	No-mild		93 (25.7)	269 (74.3)	1	
	Moderate-severe		13 (59.1)	9 (40.9)	4.18 (7.3–17.1)	**0.23**

Sedation levels	Calm or no answers		56 (21.9)	200 (78.1)	1	
	Restless		10 (31.3)	22 (68.7)	1.62 (22–38)	**0.22**
	Agitated		9 (33.3)	18 (66.7)	1.78 (2.4–3.1)	**0.24**
	Very agitated		17 (47.2)	19 (52.8)	3.19 (1.5–6.4)	**0.19**
	Combative		14 (42.4)	19 (57.6)	2.63 (1.6–8.1)	**0.21**

N.B: Bold *p*-value = significant association on bivariable logistic regression at a *p*-value< 0.25. 1-Reference group.

**Table 4:** Results of multivariable logistic regression of factors independently associated with the emergence delirium in older patients who underwent elective surgery at Teaching Hospitals of Ethiopia, Ethiopia (*n* = 384).

Variables	Categories		AOR (95% CI)
Educational status	High education		1
	Low education		1.33 (0.11–1.14)
	No		1.21 (0.03–2.58)

Marital status	Married		1
	Single		0.88 (0.19–2.33)
	Divorced		1.11 (0.37–1.66)

Comorbidity	No		1
	Yes		9.45 (0.83–22.99)

Preoperative hemoglobin levels	Nonanemic		1
	Anemic		**1.98 (1.77–3.46)**

Types of anesthesia	Regional anesthesia		1
	General anesthesia		4.33 (0.5–11.66)

Premedication	Anticholinergic	No	1
		Yes	**8.5 (6.85–17.35)**
	Opioids	No	1
		Yes	**7.89 (3.22–27.8)**

Postoperative pain	No-mild		1
	Moderate-severe		**3.10 (2.07–9.84)**

Sedation level	Calm or no answers		1
	Restless		3.11 (0.29–1.88)
	Agitated		0.80 (0.33–1.66)
	Very agitated		1.11 (0.04–18.06)
	Combative		2.75 (0.36–22.5)

N.B: Bold: Significant association on multivariable logistic regression.

## Data Availability

The data used to support the findings of the study can be obtained from the corresponding author upon reasonable request.

## Abbreviations

AOR: Adjusted odds ratio
ASA: American society of anesthesiologist
CAM: Confusion assessment method
COR: Crude odds ratio
CI: Confidence interval
Hgb: Hemoglobin
ICU: Intensive care unit
NRS: Numeric rating score
PACU: Postanesthesia care unit
RASS: Richmond agitation sedation scale
WHO: World health organization.
